# Impact of Ohmic-Assisted Decoction on Bioactive Components Extracted from Yacon (*Smallanthus sonchifolius* Poepp.) Leaves: Comparison with Conventional Decoction

**DOI:** 10.3390/molecules22122043

**Published:** 2017-11-23

**Authors:** Forough Khajehei, Mehrdad Niakousari, Maral Seidi Damyeh, Nikolaus Merkt, Wilhelm Claupein, Simone Graeff-Hoenninger

**Affiliations:** 1 Department of Agronomy, Institute of Crop Science, University of Hohenheim, Fruwirthstr. 23, Stuttgart 70599, Germany; claupein@uni-hohenheim.de (W.C.); simone.graeff@uni-hohenheim.de (S.G.-H.); 2 Department of Food Science and Technology, School of Agriculture, Shiraz University, Shiraz 71441-65186, Iran; niakosar@shirazu.ac.ir (M.N.); maralseidi@yahoo.com (M.S.D.); 3 Department of Quality of Plant Products, Institute of Crop Science, University of Hohenheim, Emil-Wolff-Straße 23, Stuttgart 70599, Germany; merkt@uni-hohenheim.de

**Keywords:** antioxidant activity, decoction, flavonoids, ohmic-assisted decoction, phenolic acids, *Smallanthus sonchifolius* Poepp., yacon leaves

## Abstract

Yacon (*Smallanthus sonchifolius* Poepp.) leaves are a potentially rich source of bioactive compounds, such as phenolic acids and flavonoids. In this study, the effect of the extraction method (ohmic-assisted decoction (OH-DE) and decoction (DE)), yacon cultivar (red and white), and leaf age (young and old) on the quality/quantity of extracted phytochemicals were investigated. Extraction yield, energy consumption, total phenolic content (TPC), total flavonoid content (TFC), ABTS and DPPH radical scavenging activity, and ferric reducing antioxidant power (FRAP) were determined. Additionally, HPLC-DAD was used to identify the major individual phenolic and flavonoid compounds of yacon leaves. The results showed that a three-way interaction of process-variables (extraction method×yacon cultivar×age of leaves) influenced the extraction yield, TPC, TFC, ABTS, and DPPH radical scavenging activity, and FRAP, significantly (*p* < 0.05). However, energy consumption of the extraction process was only affected by method of extraction (*p* < 0.05) and was halved when OH-DE was applied as compared to DE alone. Additionally, the phytochemical quality of extracts was either improved or comparable when OH-DE was used for extraction. Also, it was shown that yacon leaves contained considerable amounts of caffeic acid, chlorogenic acid, ferrulic acid, myricetin, *p*-coumaric acid, and rutin, while leaves of the red cultivar had higher contents of each compound compared to leaves of the white cultivar.

## 1. Introduction

The growing attention towards the health benefits of medicinal plants has originated from an increasing tendency of consumers towards substituting synthetic compounds in food and pharmaceutical products by their potential natural alternatives. Phenolic acid and flavonoid compounds are secondary metabolites of plants and, due to their antioxidant activity, one of the most important groups of bioactive constituents of medicinal plants. A huge number of studies has been conducted to determine the phytochemicals and in particular phenolic profiles of medicinal plants, their mechanism of action against certain diseases, health-enhancing effects, safety and their potential to be used in food products, nutraceuticals and pharmaceuticals [1,2].

Yacon (*Smallanthus sonchifolius* Poepp.) is a tuber plant that is native to the Andean region. Yacon leaves have been prepared as a traditional medicinal tea that can be useful against chronic diseases, such as diabetes and renal disorder. For this reason, yacon leaves have recently gained attention as a natural remedy. In this regard, several studies has been focusing on hypoglycaemic activity of yacon leaves [3,4,5,6]. Furthermore, Investigations have shown that yacon leaves have antioxidant, antifungal, and pesticidal properties [6,7,8]. Therefore, determination of the phytochemicals in yacon leaves extract that might induce such biological activities has been the focus of several studies. Yacon leaves contain biologically active compounds such as catechol, phenolic acids, terpenes, and flavonoids, to which their antioxidant, antidiabetic, and antitumor properties may be attributed [3,6,8]. Phytochemical content and antioxidant activity have been reported in extracts of yacon leaves cultivated in Brazil using aqueous decoction and infusion methods and their methanolic extracts [9]. The influence of genotype and solvent on phytochemicals extracted from yacon leaves cultivated in the Czech Republic was indicated in study of Russo et al. [10]. Also, different amounts of phytochemicals have been reported in yacon leaves from various origins such as Ecuador and China [11,12]. Thus, the quantities of phytochemicals determined depends on various parameters, such as the origin of the yacon leaves, method and the solvent used for extraction. More specifically, chlorogenic acid, caffeic acid, ferulic acid, quercetin, rosmarinic acid, luteolin, gallic acid, rutin, and myricetin were among the phytochemicals that have been identified in various quantities in crude extracts of yacon leaves [5,9,10,11,12,13,14].

Several methods have been developed to perform extraction of phytochemicals and their effects on composition and functionality of extracts from plant materials have been studied [15,16]. Novel technologies, such as microwave-, ultrasound-, and ohmic-assisted extraction, have been applied to improve the efficiency of extraction of phytochemicals from food materials. These methods have attracted considerable interest from the scientific community [15]. Particularly, ohmic-assisted extraction is a process where an alternating electrical current is passed directly through the processed materials. The thermal energy required for the extraction process is generated internally as a result of this passage of electrical current through the materials [17]. Ohmic heating has been the topic of a considerable number of studies to improve the extraction process in terms of efficiency and quality of product. The effectiveness of ohmic-assisted extraction of polyphenols from red grape pomace, anthocyanins from black rice bran, and phytochemicals from colored potato has been previously reported in the literature [18,19,20]. When compared to conventional extraction methods, ohmic-assisted extraction has more merits. Ohmic-assisted extraction requires a shorter processing time due to the rapid generation of heat within the plant material, it consumes less energy, and the operating costs are much lower [21]. Besides, ohmic heating technology has been evaluated as a green technology, because it works with electricity, which can be produced using renewable energies (e.g., solar and wind energy) and results in lower carbon emissions [22,23].

To the best of our knowledge, there are no studies on total phenolic and flavonoid content, antioxidant activity, and quantity of main individual phenolic and flavonoid compounds of leaves of red and white yacon cultivars which were cultivated in Germany. Therefore, the main objectives of this work were to: (1) evaluate the influence of two extraction methods, namely, ohmic-assisted decoction (OH-DE) and decoction (DE), on biological characteristics of aqueous crude extracts from yacon leaves and; (2) determine the effect of cultivar and age of yacon leaves on the polyphenol profile and antioxidant activity.

## 2. Results and Discussion

### 2.1. Extraction Process: Extraction Yield and Energy Consumption

According to previous studies, extraction of yacon leaves using the DE method and water as an environmental friendly solvent resulted in extracts with higher amounts of extracted phytochemicals [9,10]. Therefore, in this study aqueous extraction of yacon leaves was investigated using the DE method with different heating mechanisms. The DE method consists of using a hot surface from which the thermal energy is transferred to food materials. DE was used as conventional method. During DE, heat transfer occurs according to the conduction and convection principles. OH-DE was performed taking advantage of volumetric heating principles as a novel technique. 

Electrical conductivity is a key factor in ohmic heating. The addition of salt in an extraction medium can increase the electrical conductivity and ensure the generation of heat within the mixture [21,24]. Therefore, before OH-DE was used in this study, 0.3% *w*/*v* of NaCl was added to the mixture of leaves and water in order to improve the electrical conductivity of the extraction medium. According to Table 1, there were significant differences between the electrical conductivities of each sample with and without salt (*p* < 0.05). The electrical conductivity increased between 1.28 and 1.98 times the original sample when salt was added to the mixture, which can enhance the generation of thermal energy during OH-DE (Table 1).

Table 2 shows the energy consumption and the corresponding extraction yield. The energy consumption was only influenced significantly by the extraction method (*p* < 0.0001) (Table 3). When OH-DE was applied, the heating-up time (28.20 ± 8.02 min on average) was considerably lower than the heating-up time when DE was used (44.12 ± 6.98 min on average). Consequently, the energy consumption was halved using OH-DE in comparison to DE (Table 2). Rapid heat generation inside the extraction medium by conversion of electrical energy into thermal energy when using OH-DE is seen as the main reason for significant reduction of energy in contrast to DE.

In this study, extraction yield was assessed using the total dry matter of the extracts. Statistical analysis of data showed that the interaction between extraction method, cultivar, and age of yacon leaves influenced the yield of extraction significantly (*p* = 0.0339, Table 3). The extraction yield of leaves of the red cultivar was higher compared to that of the leaves of the white cultivar (Table 2). The extraction yield of old leaves of the red cultivar extracted by OH-DE (extraction yield (%) = 5.33 ± 0.11) was significantly higher than the yield of the other samples, while the lowest yield of extraction corresponded to the young leaves of the white cultivar extracted by DE (extraction yield (%) = 4.37 ± 0.33, Table 2). No significant statistical difference was observed between the extraction yields when the same kind of leaves were extracted with different methods except for the young leaves of the white cultivar, which had significantly lower yields of extraction when extracted with DE in comparison to OH-DE (Table 2).

**Figure 1:**
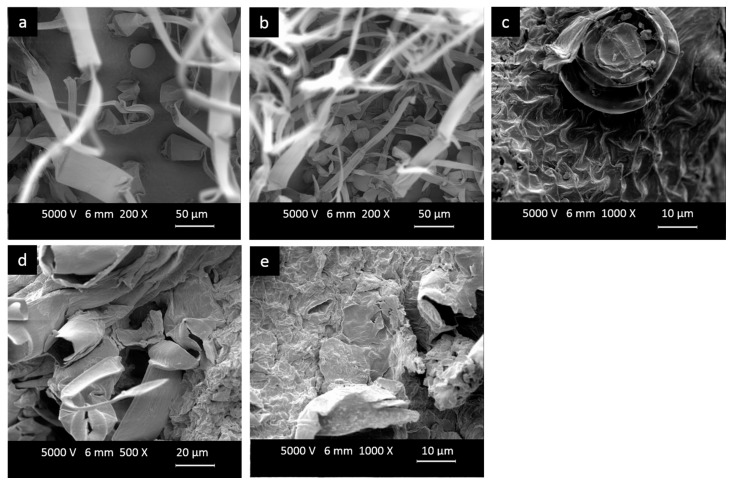
Scanned electron micrograph of young yacon leaves of the red cultivar (**a**) upper surface of fresh leaves-200×; (**b**) lower surface of fresh leaves-200×; (**c**) surface of dried leaves-1000×; (**d**) surface of dried leaves after OH-DE-500× and (**e**) surface of dried leaves after DE-1000×.

In addition, the effects of processing on the physical structure of leaves were observed to better understand the differences between mechanisms of extraction by OH-DE compared to DE. Figure 1 illustrates the effect of processing on young leaves of the red cultivar as an example.

Figure 1a,b show the glandular and non-glandular trichomes on the upper and lower surface of epidermis of a fresh leaf, respectively. Non-glandular trichomes are in the form of hairs and glandular trichomes are raised above the epidermis of the leaves. The glandular trichomes take part in the production and storage of compounds that are essential for plant adaptation and interaction with environmental conditions [6,25]. The epidermis of the leaves were intensely affected after drying, and shrinkage and collapse of leaf surface is the outcome of drying the leaves (Figure 1c). Collapse of cell structure due to drying has been previously reported [26]. Figure 1d,e can be used to distinguish the difference in effect of OH-DE and DE on the physical structure of leaves, respectively. The cell structure of leaves after OH-DE is more porous and damaged compared to the sample treated by DE (Figure 1d,e). Drastic destruction of cellular structure and consequently a release of compounds inside the cell are a result of applying ohmic heating, which has been reported by other researchers [19,22,23]. Ruptured cells are a consequence of the rapid heating mechanism of ohmic heating. Suddenly converting electrical energy into thermal energy causes heat stress to cells, which in turn reuslts in rupture in their structure, and enhances the diffusion of solutes in solvents during the extraction process. Besides, the dominant non-thermal effect of ohmic heating known as cell membrane electroporation could be another factor that can cause damage to cells which further assists in the extraction process. The breakage of cellular structure during DE extraction is less severe, because the heat transfers by means of conduction and convection takes place at a slower pace in contrast to volumetric heat transfer in the case of OH-DE [23,27]. Consequently, the average amount of solid diffused in the solvent is lower using DE in comparison to OH-DE (Table 2).

### 2.2. Total Phenolic Content (TPC)

Phenolic compounds are secondary metabolites in plants, which contribute to the sensorial (taste, flavor, color, etc.) and functional (antioxidant activity, antidiabetic, anticancer activity, etc.) characteristics of food products [2,28]. The amount of TPC in yacon leaves is shown in Table 4.

Statistical analysis of data determined the significant effect of interactions between extraction methods, cultivar, and age of leaves on TPC (*p* = 0.0483, Table 3). The average amount of TPC was higher in red cultivar leaves as compared to the white cultivar. This result is aligned with the higher average extraction yield for leaves of the red cultivar (Table 2 and Table 4). The TPC that was extracted from leaves of red cultivar varied between 59.42 ± 2.51 and 76.67 ± 21.67 (mg GAE g DW^−1^), while leaves of white cultivar contained between 46.51 ± 0.79 and 59.23 ± 14.81 (mg GAE g DW^−1^) of TPC (Table 4). The TPC of yacon leaves has also been investigated by other researchers [9,10,11,13,14]. The amount of TPC in yacon leaves extracted by DE in a study of De Andrade et al. was 42.2 ± 4.58 (mg GAE g DW^−1^) [9]. Also, the TPC in yacon aqueous extract obtained by DE from five yacon landraces varied between 17.10 ± 09 and 43.20 ± 3.20 (mg GAE g DW^−1^) [10]. Variations in the TPC content of yacon leaves reported in previous studies and in our study could be due to a combination of several factors. The quality of plant material is an important factor that can affect the extraction yield and quality of biological compounds. The quality of plant material can be influenced by several factors, such as cultivar, environmental conditions, and handling and processing conditions of plant material after harvest. Extraction method, solvent type, the ratio of solvent to plant material (*v*/*w*), particle size of plant materials, and time and temperature of extraction are some of the technological parameters that can influence the extraction of biological compounds and lead to differences in the overall TPC [29,30,31,32].

Furthermore, no significant statistical difference was indicated between the TPC of the same type of leaves, which were extracted with DE and OH-DE (Table 4). This indicates that there was no adverse effect on the amount of TPC by OH-DE. In fact, the average amount of TPC extracted from old leaves of the white yacon cultivar, young and old leaves of the red cultivar using OH-DE was 19.32%, 29.03% and 13.79% higher compared to results of extracted TPC from the same type of leaves using DE, respectively. In the case of young leaves of the white cultivar, the average amounts of TPC extracted with OH-DE and DE were statistically comparable, but the amount of extracted TPC was 9.85% lower using OH-DE in comparison to extracted TPC by DE. Therefore, OH-DE can be an effective alternative method for extraction of phenolic compounds, which supports the results of other investigators [18,19,20].

### 2.3. Total Flavonoid Content (TFC)

Flavonoids are a vast group of polyphenolic compounds, which can be found particularly in the leaves of plants. They are responsible for taste and flavor of food materials, while having certain health benefits such as antidiabetic effects and antioxidant and anticancer activity [33,34]. Table 4 shows the results for the TFC of yacon leaves. The interaction between the main process variables, namely, cultivar of yacon, age of leaves, and method of extraction, on TFC was significant (*p* = 0.0484, Table 3). Similar to the TPC outcomes, the average amount of TFC of leaves of red cultivars were higher than that of leaves of the white cultivar (Table 4). The average amount of TFC in leaves of the red cultivar ranged from 134.01 ± 12.99 to 199.29 ± 58.75 (mg RE g DW^−1^), while it ranged from 110.34 ± 4.68 to 153.79 ± 40.64 (mg RE g DW^−1^) in leaves of the white cultivar (Table 4). Although, no significant difference was determined between mean values of TFC in the same type of leaves extracted with different methods (Table 4), The average amount of TFC of leaves with the same age and cultivar extracted by OH-DE was higher than that of the same leaves extracted using DE. However, there were some exceptions in cases with young leaves from the white cultivar (Table 4).

TFC of yacon leaves in aqueous extracts obtained using DE was reported at 39.72 ± 1.37 (mg RE g DW^−1^) in the study of De Andrade et al., which is considerably lower than our findings [9]. Similar to TPC, the amount of extracted TFC can be affected by various factors such as different ratios of solvent to plant material, extraction condition, origin, cultivar, quality, and particle size of the leaves. Moreover, in the present study, each sample was extracted two times, while in the study of De Andrade et al. each sample was extracted only once [9]. According to principles of mass transfer, renewal of solvent can keep the concentration gradient high and postpone reaching the equilibrium point. Therefore, adding fresh water to leaves for a second cycle of extraction as done in this study might be a contributing factor to higher extracted yields of flavonoid compounds.

### 2.4. Antioxidant Activity

#### 2.4.1. ABTS Radical Scavenging Activity

ABTS radical scavenging activity is a fast and simple method to determine the total antioxidant capacity in food materials which is determined by measuring the reduction in blue-green color of the radical cation ABTS through donation of hydrogen or electron by the antioxidant compounds [35].

The ABTS radical scavenging activity of yacon leaves are presented in Table 4. According to the statistical analysis of data, the interaction effect of the three process variables (extraction method, cultivar, and age of leaves) had a significant effect on the ABTS radical scavenging activity of leaves (*p* = 0.0055, Table 4). Higher ABTS radical scavenging activity of red yacon leaves in comparison with that of the white yacon leaves aligns with their higher TPC and TFC results. The ABTS radical scavenging activity of old leaves of the red cultivar that was extracted with OH-DE (2378.89 ± 52.70 (mM TE g DW^−1^)) was significantly higher than that of all other samples (Table 4). ABTS radical scavenging activity of aqueous extracts of yacon leaves extracted by decoction was reported at 391.55 ± 22.32 (µM Trolox g DW^−1^) in a study of De Andrade et al., which is significantly lower than the results obtained in this study [9]. Variation in cultivar of yacon leaves and differences in origin of them might be among contributing factors to different ABTS radical scavenging activity obtained in this study. Furthermore, as phenolic acid and flavonoid compounds may contribute to ABTS radical scavenging activity of yacon leaves, the higher amount of ABTS radical scavenging activity obtained in the present study is in agreement with higher amounts of TFC and TPC extracted compared to findings of De Andrade et al. [9]. ABTS radical scavenging activity of aqueous extracts of yacon leaves has been also studied using EC_50_ methodology in study of Sugahara et al. which cannot be compared to the results of present study due to differences in analytical methods [36].

ABTS radical scavenging activity of yacon leaves indicated a moderate correlation with TPC (*R* = 0.452), while it did not significantly correlate with TFC (Table 5). Antioxidant activity of phenolic acid and flavonoid compounds and their mechanism of action in regards to free radicals are related to their structure. Therefore, differences in correlation among TFC and TPC with the result of ABTS radical scavenging activity might be under influence of the structure of phenolic and flavonoid compounds in yacon leaves extract [37,38].

#### 2.4.2. DPPH Radical Scavenging Activity

DPPH radical scavenging activity method is widely used to determine the ability of antioxidants in a sample to quench free radicals of DPPH by donating hydrogen. DPPH radicals have a purple color, which undergoes a color change upon neutralization when it receives hydrogen [35]. 

The result of evaluating the DPPH radical scavenging activity of yacon leaves is reported in Table 4. The statistical analysis showed that the interaction between the three independent process variables (extraction method, cultivar, and age of leaves) significantly influenced the DPPH radical scavenging activity of leaves (*p* = 0.0034) (Table 3). The red yacon leaves showed higher average values of DPPH radical scavenging activity in comparison to the leaves of the white cultivar (Table 4). This is in agreement with results previously reported in a study of Russo et al., which showed higher DPPH radical scavenging activity in yacon leaves that belonged to landraces that had tubers with purple-grey colored peels compared to landraces with red-purple and grey-orange colored tuber peels [10]. Likewise, the outcome of ABTS radical scavenging activity the old leaves of the red cultivar extracted with OH-DE showed a significantly higher DPPH radical scavenging activity than the other samples (Table 4). The result of Pearson correlation analysis showed that there was a moderately positive correlation between the DPPH radical scavenging activity and TPC (*R* = 0.545, Table 5). Also, a strong positive correlation was determined between ABTS radical scavenging activity and DPPH radical scavenging activity (*R* = 0.983, Table 5). In addition, there was a week positive correlation between DPPH radical scavenging activity and TFC (*R* = 0.350, Table 5). DPPH radical scavenging activity of yacon leaves extract was studied according to EC_50_ methodology by other researches [9,36]. Due to differences in analytical methods applied in these investigations and the present study, the results cannot be compared. A positive correlation between TPC and TFC and DPPH radical scavenging activity of yacon leaves extract was reported in the study of De Andrade et al., which is supported by our findings [9]. This suggests that the antioxidant characteristics of yacon leaves are attributable to phenolic acids and flavonoid compounds.

#### 2.4.3. FRAP Assay

FRAP assay is another method based on electron transfer for measuring the antioxidant characteristics of food materials. In this method, an antioxidant’s power is determined under acidic conditions by reducing the ferric 2,4,6-tripyridyl-s-triazine complex to the ferrous complex. The later exhibits an intense blue color which can be measured spectrophotometrically. The FRAP assay results are reported as an equivalent concentration of ferrous ions (mM) [35].

The FRAP of yacon leaves is noted in Table 5. The statistical analysis of data showed that the FRAP of yacon leaves was significantly affected by the interaction between extraction method, cultivar, and age of leaves (*p* = 0.0084) (Table 3). In line with the DPPH and ABTS radical scavenging activity outcomes, the leaves of the red cultivar showed a higher average amount of FRAP in comparison to leaves of the white cultivar (Table 4). Young and old leaves of the red cultivar, which were extracted with OH-DE had the highest average amount of FRAP (994.55 ± 83.38 and 976.90 ± 56.23 (mM Fe^2+^ g DW^−1^), respectively), were statistically comparable to each other (Table 4). The results of present study are in agreement with results of the study of Russo et al. who reported higher FRAP values for the extract that was obtained from leaves of yacon samples with grey-purple tuber peel [10]. Moreover, a positive correlation (*R* between FRAP and TPC = 0.627, *R* between FRAP and TFC = 0.542, *R* between FRAP and DPPH radical scavenging activity = 0.791, *R* between FRAP and ABTS radical scavenging activity = 0.715) was determined between FRAP of yacon leaves and their TPC, TFC, DPPH radical scavenging activity, and ABTS radical scavenging activity (Table 5). The correlation between TPC or TFC and FRAP was stronger than the correlation between TPC or TFC and DPPH radical scavenging activity or ABTS radical scavenging activity (Table 5). This might suggest that: (1) the phenolic and flavonoid compounds in yacon leaves express better antioxidant activity in acidic conditions and (2) the mechanism of action for dominant antioxidant compounds in yacon leaves is not based on the radical quenching mechanism, but instead is in line with their ability to act as reductants.

### 2.5. Individual Phenolic acid and Flavonoid Compounds

In the present study, HPLC-DAD was used for quali-/quantification of phenolic compounds in yacon leaves. Table 6 reports the amount of six phenolic compounds, which were successfully identified and quantified using external standards, including four hydroxycinnamic acids, namely, ferrulic acid, caffeic acid, *p*-coumaric acid, chlorogenic acid, and two flavonoid compounds, namely, myricetin and rutin. The presence of ferrulic acid, caffeic acid, *p*-coumaric acid, chlorogenic acid, myricetin and rutin in aqueous extract of yacon leaves has been confirmed according to existing literature [5,9,10,11,13,14].

The outcomes (Table 6) showed that caffeic acid had the highest average amount, followed by chlorogenic acid, and *p*-coumaric acid, while ferrulic acid had the lowest average amount among the hydroxycinnamic acids in yacon leaves. Myricetin and rutin were the two flavonoid compounds which were detected in yacon leaves in this work. The average amount of myrecitin was much higher than the mean amount of rutin constitution of yacon leaves (Table 6). The findings showed that the average amount of each individual phenolic and flavonoid compound, which was screened in this study, was higher in the leaves of the red cultivar when compared to the leaves of the white cultivar. This aligns with the results of TPC and TFC and antioxidant activity measurements (Table 4 and Table 6). Furthermore, these results are in agreement with the outcomes of the study of Russo et al., who reported high amounts of phenolic compounds in aqueous extracts of yacon leaves, while there was a difference between the profile of phenolic compounds and their quantity in extracts of leaves from different yacon landraces [10].

In addition, the highest amount of each phenolic and flavonoid compound was obtained when young leaves of the red yacon cultivar were extracted by OH-DE. Old leaves of red yacon extracted with OH-DE ranked second with higher individual phenolic acids and flavonoids (Table 6). Also, the average amount of myricetin, *p*-coumaric acid, and rutin was higher in leaves of the white cultivar when OH-DE was applied for extraction (Table 6). However, the average amount of caffeic acid and chlorogenic acid extracted from leaves of the white cultivar was higher when the extraction was performed by DE (Table 6). Overall, the results showed no difference in profile of identified individual phenolic acids and flavonoid compounds which were extracted from yacon leaves with exception for ferrulic acid (Table 6). Ferullic acid was only extracted from young leaves of the white cultivar when OH-DE was applied for extraction (Table 6). Higher retention of phenolic compounds, when ohmic heating was applied, has been reported by other researchers in cases of extraction of polyphenols from red grape pomace, extraction of colorant from rice bran, and extraction of phytochemicals from potato [18,19,20]. Rapid heating, when ohmic heating is applied, reduces the overall processing time by reducing the heating up time. Shorter overall processing time might play a role in producing less destructive effects on heat sensitive phenolic compounds. Furthermore, rapid heating can cause structural damage to cellular structures through heat stress, as discussed in previous sections and enhance the release of phenolic compounds. In addition, electroporation effect of ohmic heating can be named as another contributing factor in release of higher amounts of biological compounds from cellular structures [21,39].

## 3. Materials and Methods

### 3.1. Chemicals

Ascorbic acid, Folin–Ciocalteu’s reagent, FeCl_3_, FeSO_4_, NaOH, HCl, and NaNO_2_, were purchased from Merck (Darmstadt, Germany). 2,4,6-Tris(2-pyridyl)-1,3,5-triazine (TPTZ) and 2,2′-azino-bis(3-ethylbenzothiazoline-6-sulfonic acid) diammonium salt (ABTS), were provided from Sigma (Darmstadt, Germany). Gallic acid (Scharlau, Barcelona, Spain), 2,2-diphenyl-1-picrylhydrazyl (DPPH) (CalBiochem, Darmstadt, Germany), AlCl_3_ (Fluka, Seelze, Germany), Na_2_CO_3_ (AppliChem, Darmstadt, Germany), potassium persulfate (Bernd Kraft, Duisburg, Germany), Trolox (Cayman, Ann Arbor, MI, USA), were used. Caffeic acid, myricetin, *p*-coumaric acid, and quercetin (HPLC grade) were purchased from Sigma (Darmstadt, Germany). Ferrulic acid, gallic acid, kaempherol, and rutin (HPLC grade) were supplied from Carl Roth GmbH (Karlsruhe, Germany). Methanol and ethanol from Chemsolute (Hamburg, Germany), acetic acid (AppliChem, Darmstadt, Germany), and acetonitrile (J.T.Baker, Hamburg, Germany) purchased were HPLC grade.

### 3.2. Plant Material

Young and old yacon leaves of two different yacon cultivars (red and white) were collected from a field trial carried out at the organically operating Kleinhohenheim research station of the University of Hohenheim (Stuttgart, Germany) in October 2015 at harvest time. Cultivars of yacon were classified according to the color of their tuber peels. Yacon plants were grown at the same field under the same growing conditions and management to ensure *ceteris paribus*. Young leaves refer to smaller leaves on top of yacon stems, while old leaves imply big leaves collected from the lower part of stems. On both cultivars, the number of leaves was counted to ensure that the collected leaves had the same growth stage. Collected leaves were dried at 40 °C for 24 h. Afterwards, the leaves were kept in a dry and cool place for further analysis. Dried leaves were ground and passed through a sieve (40 mesh) to have homogenous samples, before initiating the extraction process.

### 3.3. Extraction Process

#### 3.3.1. Decoction (DE)

Leaf powder was mixed with distilled water (ratio of leaves:water = 1:20 (*w*:*v*)). Then, the mixture was heated to boiling point under cooling reflux to avoid water loss with evaporation. The heating up time was recorded using a stopwatch. Afterwards, holding time at boiling point was 10 min. Then, the extract was cooled and filtered through Whatman No. 40 paper (Whatman, Buckinghamshire, UK). The residue was extracted under the same conditions for the second cycle of extraction. The extracts were mixed together and evaporated by means of a vacuum rotary evaporator at 35 °C (Rotavapor^®^ R-100, Büchi, Essen, Germany) and freeze dried.

#### 3.3.2. Ohmic-Assisted Decoction (OH-DE)

OH-DE was performed using an ohmic device (designed and built in the Transport Properties Laboratory at the Department of Food Science and Technology, Shiraz University, Iran) that consisted of a Teflon cylindrical chamber (7 cm internal diameter and 25 cm length), which has two titanium-coated 316 stainless steel electrodes. The device is automated so the voltage (0–350 V), current (0–16 A), and temperature could be monitored.

Prior to the extraction process with OH-DE, leaf powder was soaked for 10 min in salted water (0.3% *w*/*v* NaCl solution) (ratio of leaves:water = 1:20 (*w*:*v*)). OH-DE was performed using 150 V under a cooling reflux in order to avoid losing water through evaporation. Heating up time was also recorded. Then, OH-DE was maintained for 10 min holding time after reaching the boiling point. Afterwards, the extract was filtered through Whatman No. 40 paper (Whatman). The residue was extracted under the same conditions for a second cycle and extracts were mixed together, evaporated using a Rotavapor^®^ R-100 vacuum rotary evaporator at 35 °C, and freeze dried.

### 3.4. Yield of Extraction

To evaluate yield of extraction, total solid contents of extracts were measured gravimetrically. Approximately five grams of extracts were dried at 60 ± 1 °C overnight and then cooled in a desiccator for an hour before weighing. Yield was calculated using Equation (1):

Yield % = ((weight of sample after dehydration)/(initial weight of sample)) × 100(1)

### 3.5. Measurement of Electrical Conductivity

The electrical conductivity of plant materials was evaluated by an electrical conductivity meter (Mi180, Milwaukee, Szeged, Hungary). Average electrical conductivity in the ratio of 1:20 (leaves:water) at room temperature was recorded, while one sample contained 0.3% *w*/*v* NaCl and another sample was without NaCl.

### 3.6. Energy Consumption

Energy consumption was evaluated using a digital single phase kWh meter with 0.01 kWh accuracy. The device was connected to the main power cable of the ohmic device and mantel. The total energy consumption was calculated by summing up the energy consumption of both stages of extraction for each extraction experiment.

### 3.7. Total Phenolic Content (TPC)

Briefly, 1 mL of the reconstituted yacon extract (1 mg mL^−1^ of distilled water) was added to 1 mL of Folin–Ciocalteu’s reagent. After 3 min, 1 mL of saturated Na_2_CO_3_ (35%) was added to the mixture. Then, the volume of mixture was made up to 10 mL with distilled water. Afterwards, the reaction mixture was left in darkness for 90 min. The absorbance was read at 725 nm using UV/Visible spectrophotometer (Ultrospec 3100 Pro, Amersham Bioscience, Buckinghamshire, UK). The calibration curve was generated with gallic acid solution (0.004–0.25 mg gallic acid mL^−1^ distilled water) as a reference standard. TPC is expressed as gallic acid equivalent per gram of dried weight of leaves (mg GAE g DW^−1^) [40].

### 3.8. Total Flavonoid Content (TFC)

To measure the TFC, 500 µL of the reconstituted yacon extract (1 mg mL^−1^ of distilled water) was added to 1 mL of NaNO_2_ (5%) and mixed well. After 6 min, 1 mL of 10% AlCl_3_ and 10 mL of NaOH (1 M) were added to the mixture and the volume of mixture was adjusted to 25 mL with distilled water. Afterwards, the reaction mixture was left to stand for 15 min at room temperature before reading the absorbance at 510 nm by means of UV/Visible spectrophotometer (Ultrospec 3100 Pro, Amersham Bioscience). Rutin (0.06–4 mg rutin mL^−1^ 70% ethanol) was used as a reference standard to draw the standard curve. TFC was expressed as rutin equivalent per gram of dry weight of leaves (mg RE g DW^−1^) [41].

### 3.9. Determination of Antioxidant Activity

#### 3.9.1. ABTS (2,2′-Azino-bis(3-ethylbenzothiazoline-6-sulfonic Acid) Diammonium Salt) Radical Scavenging Assay

ABTS was dissolved in water (7 mM concentration). Then, potassium persulphate (2.45 mM) was added to ABTS solution (1:1, *v*/*v*) and the mixture was left to stand in the dark at room temperature for 12–16 h before being used to produce ABTS radical cations (ABTS^•+^). The ABTS^•+^ solution was diluted with distilled water to an absorbance of 0.70 ± 0.02 at 734 nm. The %-inhibition of extract against ABTS^•+^ solution was performed as following [42]: 3.0 mL of diluted ABTS^•+^ solution was briefly added to 100 µL of leaves extract (1 mg mL^−1^). The reaction solution was kept in 30 °C after mixing for 10 min. The absorbance was read at 734 nm with UV/Visible spectrophotometer (Ultrospec 3100 Pro, Amersham Bioscience). A blank was prepared using distilled water. The %-inhibition of leaves extract and Trolox solutions (0.02–0.2 Trolox (mM)) which were used as the reference standard for generation of standard curve was calculated using Equation (2):

Inhibition (%) = ((A_B_ − A_S_)/A_B_) × 100 (2)

where A_B_ is the absorbance of the blank sample and A_S_ is the absorbance of samples. ABTS radical scavenging activity was expressed as Trolox equivalent per gram of dried weight of leaves (mM TE g DW^−1^).

#### 3.9.2. DPPH (2,2-Diphenyl-1-picrylhydrazyl) Radical Scavenging Activity

The DPPH radical scavenging activity of yacon leaves extracts was measured as follows [42]: 0.1 mL of the reconstituted yacon extract (1 mg mL^−1^ of distilled water) was added to 3 mL of freshly prepared 6 × 10^−5^ M methanolic DPPH^•^ solution. Then, the mixture was kept at 37 °C for 20 min. The absorbance was read at 515 nm using UV/Visible spectrophotometer (Ultrospec 3100 Pro, Amersham Bioscience). Distilled water was used for preparation of the blank sample. The %-inhibition was calculated according to Equation (2). Calibration curve was drawn for %-inhibition of ascorbic acid solution (0.02–0.2 mg ascorbic acid mL^−1^ distilled water) as a reference standard. DPPH radical scavenging activity was expressed as mg ascorbic acid equivalent per gram of dried weight of leaves (mg AAE g DW^−1^).

#### 3.9.3. Ferric Reducing Antioxidant Power (FRAP) Assay

The FRAP working solution was prepared by mixing 300 mM acetate buffer (pH 3.6), 10 mM TPTZ (2,4,6-Tris(2-pyridyl)-1,3,5-triazine) in HCl (10 mM), and 20 mM FeCl_3_ solution in a 10:1:1 (*v*/*v*/*v*) ratio. 0.15 mL of the reconstituted yacon extract (1 mg mL^−1^ of distilled water) was mixed with 2.85 mL FRAP solution and incubated at 37 °C for 30 min. The total antioxidant activity of the samples was evaluated by the absorbance of Fe^2+^-TPTZ at 593 nm using UV/Visible spectrophotometer (Ultrospec 3100 Pro, Amersham Bioscience). The results of the FRAP assay were expressed in FeSO_4_ (mM) equivalent per gram dry weight of leaves (mM Fe^2+^ g DW^−1^) [43].

### 3.10. Measurement of Individual Phenolic Acid and Flavonoid Compounds by Means of HPLC

High performance liquid chromatography was used for screening phenolic in yacon aqueous extracts. A Merck-Hitachi HPLC system (HPLC, Darmstadt, Germany) operated using an L-7100 solvent delivery pump, an L-7200 auto-sampler, a Smartline column oven, an L-7612 solvent degasser, and DAD L-7450A detector. Separation of phenolic compounds was performed using a Kinetex 5 μ 00G-4601 E0 column (Phenomenex, Torrance, CA, USA)) while it was kept at a constant 25 °C. Data was analyzed using D-7000 HSM software (Merck-Hitachi, Darmstadt, Germany). Mobile phase, consisting of A (acetic acid (2%)) and B (acetic acid (0.5%)–acetonitrile (50:50, *v*:*v*)), was eluted gradiently as follows for a total time of 65 min: 0 min (82% A + 18% B); 25 min (75% A + 25% B); 55 min (45% A + 55% B); 56 min (0% A + 100% B); and 62 min (82% A + 18% B). An injection volume of 50.0 (μL) and flow rate of 1 (mL/min) was applied. The detector used wavelengths between 220 and 600 nm for detection. The calculated wavelength was 256 nm. The phenolic compounds in yacon extracts were identified and quantified by means of comparing the retention times and peak area equivalent standards. The following standards were used: caffeic acid, ferrulic acid, gallic acid, kaempherol, myricetin, *p*-coumaric acid, rutin, and quercetin.

### 3.11. Scanning Electron Microscopy (SEM)

SEM images of dried leaves were obtained from fresh leaves, leaves after the drying and grinding process, and extracted leaves. Dried leaves were fixed on an aluminum sample holder and spattered with 20% gold and 80% palladium for 8 min. Then, the samples were scanned using a scanning electron microscope (SEM) (DSM-940, Zeiss, München, Germany) under high-vacuum conditions with an accelerating voltage of 5.0 kV.

### 3.12. Statistical Analysis

Extraction experiments were performed in triplicate. Chemical analysis (TPC, TFC, ABTS and DPPH radical scavenging activity and FRAP) of extracts were done in duplicate in the laboratory. For HPLC analysis, one independent extract powder from each combination of variables (extraction method, leaves cultivar, and leaves age) was chosen randomly, two times independently reconstituted and two independent injections were applied. Results were reported as mean value ± standard deviation and subjected to three-way analysis of variance (ANOVA) and the mean differences between evaluated parameters were established by performing Tukey’s test at 5% significance level. Correlations between TPC, TFC, DPPH radical scavenging activity, ABTS radical scavenging activity, and FRAP results were examined using Pearson’s correlation coefficient (r). Statistical analysis of data was performed using SAS Software, version 9.4 (SAS Institute Inc., Cary, NC, USA).

## 4. Conclusions

The outcome of this work showed that utilization of OH-DE for the extraction of phytochemicals from yacon leaves offers certain benefits over DE. The average amount of extracted total phenolic and flavonoid compounds as well as antioxidant characteristics of leaves, which were processed by OH-DE, was either comparable or higher than the results obtained when the DE method was used. The energy consumption of OH-DE was also significantly lower than that of DE. Furthermore, leaves of the red yacon cultivar possessed higher levels of phytochemicals than the leaves of the white cultivar. Young leaves of red cultivar had the highest average amount of caffeic acid, myricetin, *p*-coumaric acid, rutin, and chloregic acid. Moreover, the old leaves of the red cultivar possessed the highest antioxidant activity level, contained higher average amounts of ferrulic acid, and comparable amounts of myricetin, TPC and TFC compared with young leaves of the red cultivar extracted with OH-DE. Therefore, extraction of both young and old leaves of the red cultivar by OH-DE can be suggested to achieve higher extraction of phytochemicals with lower energy consumption.

Further studies with regard to the optimization of the OH-DE process using various holding times and various voltages can be suggested. In addition, fractionation of phenolic acids and flavonoid compounds extracted from yacon leaves to determine their mechanism of action as antioxidant and/or antidiabetic compounds are required. Also, encapsulation of yacon leaves extract to maintain its health promoting effects and optimizing its application in food products, nutraceuticals and pharmaceuticals would be of interest.

## Figures and Tables

**Table 1:** Electrical conductivity of dried yacon leaves.

Cultivar	Age of Leaves	Average Electrical Conductivity (s/m)	
		With 0.3% *w*/*v* NaCl	Without NaCl
white	young	1.00 ^Aa^ ± 0.00	0.44 ^Ab^ ± 0.01
white	old	0.99 ^ABa^ ± 0.00	0.39 ^Cb^ ± 0.01
red	young	0.98 ^Ba^ ± 0.01	0.41 ^ABb^ ± 0.00
red	old	0.91 ^Ca^ ± 0.01	0.31 ^Db^ ± 0.01

Reported values are presented as mean values ± standard deviation. Mean values with the same capital letter in a column and same lowercase letter in a row are not significantly different as indicated by Tukey’s test (*p* < 0.05).

**Table 2:** Yield of extraction (%) and energy consumption.

Cultivar	Age of Leaves	Extraction Method	Yield of Extraction (%)	Energy Consumption (kWh)
white	young	OH-DE	4.84 ^AB^ ± 0.18	0.20 ^A^ ± 0.00
white	young	DE	4.37 ^C^ ± 0.33	0.43 ^B^ ± 0.05
white	old	OH-DE	5.05 ^AB^ ± 0.16	0.20 ^A^ ± 0.00
white	old	DE	5.03 ^AB^ ± 0.22	0.43 ^B^ ± 0.05
red	young	OH-DE	5.23 ^AB^ ± 0.12	0.20 ^A^ ± 0.00
red	young	DE	5.09 ^AB^ ± 0.28	0.50 ^B^ ± 0.10
red	old	OH-DE	5.33 ^A^ ± 0.11	0.20 ^A^ ± 0.00
red	old	DE	5.09 ^AB^ ± 0.25	0.46 ^B^ ± 0.11

Reported values are presented as mean values ± standard deviation. Mean values with the same capital letter in a column are not significantly different as indicated by Tukey’s test (*p* < 0.05). OH-DE = Ohmic assisted decoction and DE= decoction.

**Table 3:** ANOVA of results of energy consumption, yield of extraction, total phenolic content (TPC), total flavonoid content (TFC), DPPH radical scavenging activity (DPPH), ABTS radical scavenging activity (ABTS), and Ferric reducing antioxidant power (FRAP) as a function of extraction method (ohmic assisted decoction and decoction), cultivar (red and white) and age (young and old)of leaves.

Process-Variable	Energy Consumption	Yield of Extraction	TPC	TFC	DPPH	ABTS	FRAP
Extraction method	*p* < 0.0001	*p* = 0.0011	*p* = 0.0190	*p* = 0.029	*p* < 0.0001	*p* < 0.0001	*p* < 0.0001
Cultivar	*p* = 0.3322	*p* < 0.0001	*p* = 0.0002	*p* = 0.0016	*p* < 0.0001	*p* < 0.0001	*p* = 0.2236
Age of leaves	*p* = 0.7432	*p* = 0.0004	*p* = 0.1101	*p* = 0.0007	*p* < 0.0001	*p* < 0.0001	*p* = 0.1025
Extraction method * Cultivar	*p* = 0.3322	*p* = 0.6922	*p* = 0.0639	*p* = 0.1093	*p* = 0.1343	*p* = 0.1074	*p* = 0.0885
Extraction method * age of leaves	*p* = 0.7432	*p* = 0.1667	*p* = 0.6238	*p* = 0.5590	*p* = 0.0395	*p* = 0.0413	*p* = 0.2565
Cultivar * age of leaves	*p* = 0.7432	*p* = 0.0033	*p* = 0.8614	*p* = 0.4544	*p* = 0.0020	*p* = 0.0002	*p* = 0.4736
Extraction method * Cultivar * age of leaves	*p* = 0.7432	*p* = 0.0339	*p* = 0.0483	*p* = 0.0484	*p* = 0.0034	*p* = 0.0055	*p* = 0.0084

* Interaction between process-variables.

**Table 4:** Total phenolic content, total flavonoid content, ABTS radical scavenging activity, DPPH radical scavenging activity and ferric reducing antioxidant power (FRAP) values of yacon leaves which were extracted with ohmic-assisted decoction (OH-DE) and decoction (D).

Cultivar	Age of Leaves	Extraction Method	Total Phenolic Content (mg GAE g DW^−1^)	Total Flavonoid Content (mg RE g DW^−1^)	ABTS Radical Scavenging Activity (mM TE g DW^−1^)	DPPH Radicals Scavenging Activity (mg AAE g DW^−1^)	FRAP (mM Fe^2+^ g DW^−1^)
white	young	OH-DE	53.39 ^BC^ ± 1.94	138.16 ^B^ ± 5.12	1573.02 ^C^ ± 97.87	102.77 ^C^ ± 4.44	825.03 ^BC^ ± 22.62
white	young	DE	59.23 ^ABC^ ± 14.81	153.79 ^AB^ ± 40.64	1529.84 ^C^ ± 257.22	99.71 ^C^ ± 16.76	838.14 ^ABC^ ± 185.46
white	old	OH-DE	55.50 ^BC^ ± 10.10	135.78 ^B^ ± 16.23	2008.80 ^B^ ± 146.94	124.82 ^B^ ± 8.86	990.36 ^AB^ ± 76.61
white	old	DE	46.51^C^ ± 0.79	110.34 ^B^ ± 4.68	1560.27 ^C^ ± 87.30	94.81 ^C^ ± 5.25	798.11 ^C^ ± 52.23
red	young	OH-DE	76.67 ^A^ ± 21.67	199.29 ^A^ ± 58.75	1843.76 ^B^ ± 141.16	120.11 ^B^ ± 9.33	994.55 ^A^ ± 83.38
red	young	DE	59.42 ^ABC^ ± 2.51	157.43 ^AB^ ± 15.307	1432.31 ^C^ ± 151.37	93.18 ^C^ ± 9.88	771.17 ^C^ ± 80.00
red	old	OH-DE	67.89 ^AB^ ± 1.91	153.23 ^AB^ ± 7.56	2378.89 ^A^ ± 52.70	143.148 ^A^ ± 2.86	976.90 ^AB^ ± 56.23
red	old	DE	59.66 ^ABC^ ± 4.19	134.013 ^B^ ± 12.99	2034.06 ^B^ ± 96.17	121.28 ^B^ ± 5.87	838.40 ^ABC^ ± 75.46

Reported values are presented as mean values ± standard deviation. Mean values with the same capital letter in a column are not significantly different as indicated by Tukey’s test (*p* < 0.05). GAE = galic acid equivalent, RE = rutin equivalent, TE = trolox equivalent and AAE = ascorbic acid equivalent.

**Table 5:** Correlation coefficient (*R*) between total phenolic content, total flavonoid content, ABTS radical scavenging activity, DPPH radical scavenging activity, and ferric reducing antioxidant power (FRAP) values of yacon leaves which were extracted with ohmic-assisted decoction (OH-DE) and decoction (D) values of yacon leaves.

	Total Flavonoid Content	DPPH Radical Scavenging Activity	ABTS Radical Scavenging Activity	FRAP
DPPH radical scavenging activity	0.350 *			
ABTS radical scavenging activity	0.226 ^NS^	0.983 ***		
FRAP	0.542 ***	0.791 ***	0.715 ***	
Total phenolic content	0.943 ***	0.545 ***	0.452 **	0.627 ***

* *p* < 0.05, ** *p* < 0.01, *** *p* < 0.001, and ^NS^ not significant.

**Table 6:** Individual phenolic acid and flavonoid compounds of yacon leaves that were extracted with ohmic-assisted decoction (OH-DE) and decoction (D).

Cultivar	Age of Leaves	Extraction Method	Ferrulic Acid (mg g DW^−1^)	Caffeic Acid (mg g DW^−1^)	Myricetin (mg g DW^−1^)	*P*-Coumaric Acid (mg g DW^−1^)	Rutin (mg g DW^−1^)	Chlorogenic Acid (mg g DW^−1^)
white	young	OH-DE	0.44 ^C^ ± 0.04	17.92 ^C^ ± 0.77	24.53 ^A^ ± 3.35	3.35 ^B^ ± 0.14	0.15 ^CD^ ± 0.04	9.92 ^EF^ ± 0.54
white	young	DE	nd	19.42 ^C^ ± 0.28	16.24 ^A^ ± 11.30	2.68 ^B^ ± 1.55	0.14 ^D^ ± 0.04	15.73 ^BC^ ± 0.32
white	old	OH-DE	nd	16.81 ^C^ ± 1.35	12.01 ^A^ ± 0.32	3.37 ^B^ ± 0.32	0.25 ^BCD^ ± 0.01	9.12 ^F^ ± 0.71
white	old	DE	nd	19.94 ^C^ ± 1.11	16.85 ^A^ ± 0.92	2.94 ^B^ ± 0.03	0.16 ^CD^ ± 0.006	12.45 ^DE^ ± 0.07
red	young	OH-DE	0.70 ^B^ ± 0.04	28.95 ^A^ ± 0.21	25.98 ^A^ ± 6.31	6.18 ^A^ ± 0.05	0.52 ^A^ ± 0.01	22.33 ^A^ ± 0.68
red	young	DE	0.62 ^BC^ ± 0.11	25.11 ^B^ ± 0.69	15.03 ^A^ ± 2.40	6.03 ^A^ ± 0.17	0.42 ^AB^ ± 0.006	18.76 ^B^ ± 0.14
red	old	OH-DE	0.90 ^A^ ± 0.09	24.33 ^B^ ± 3.85	24.35 ^A^ ± 7.32	4.01 ^B^ ± 1.12	0.31 ^BCD^ ± 0.19	13.96 ^CD^ ± 3.21
red	old	DE	0.67 ^B^ ± 0.09	19.56 ^C^ ± 0.46	19.32 ^A^ ± 2.23	2.99 ^B^ ± 0.08	0.30 ^BCD^ ± 0.01	11.11 ^DEF^ ± 0.94

Reported values are presented as mean values ± standard deviation. Mean values with the same capital letter in a column are not significantly different as indicated by Tukey’s test (*p* < 0.05).
